# Membrane Lipid Composition of the Moderately Thermophilic Ammonia-Oxidizing Archaeon “*Candidatus* Nitrosotenuis uzonensis” at Different Growth Temperatures

**DOI:** 10.1128/AEM.01332-19

**Published:** 2019-10-01

**Authors:** Nicole J. Bale, Marton Palatinszky, W. Irene C. Rijpstra, Craig W. Herbold, Michael Wagner, Jaap S. Sinninghe Damsté

**Affiliations:** a NIOZ Royal Institute for Sea Research, Department of Marine Microbiology and Biogeochemistry, and Utrecht University, Texel, The Netherlands; b Division of Microbial Ecology, Centre for Microbiology and Environmental Systems Science, University of Vienna, Vienna, Austria; c Center for Microbial Communities, Department of Chemistry and Bioscience, Aalborg University, Aalborg, Denmark; d Faculty of Geosciences, Department of Earth Sciences, Utrecht University, Utrecht, The Netherlands; Kyoto University

**Keywords:** “*Ca*. Nitrosotenuis uzonensis,” lipid, GDGT, temperature, thermophile, *Thaumarchaeota*

## Abstract

“*Candidatus* Nitrosotenuis uzonensis” is the only cultured moderately thermophilic member of the thaumarchaeotal order *Nitrosopumilales* (NP) that contains many mesophilic marine strains. We examined its membrane lipid composition at different growth temperatures (37°C, 46°C, and 50°C). Its lipids were all membrane-spanning glycerol dialkyl glycerol tetraethers (GDGTs), with 0 to 4 cyclopentane moieties. Crenarchaeol (cren), the characteristic thaumarchaeotal GDGT, and its isomer (crenʹ) were present in high abundance (30 to 70%). The GDGT polar headgroups were mono-, di-, and trihexoses and hexose/phosphohexose. The ratio of glycolipid to phospholipid GDGTs was highest in the cultures grown at 50°C. With increasing growth temperatures, the relative contributions of cren and crenʹ increased, while those of GDGT-0 to GDGT-4 (including isomers) decreased. TEX_86_ (tetraether index of tetraethers consisting of 86 carbons)-derived temperatures were much lower than the actual growth temperatures, further demonstrating that TEX_86_ does not accurately reflect the membrane lipid adaptation of thermophilic *Thaumarchaeota*. As the temperature increased, specific GDGTs changed relative to their isomers, possibly representing temperature adaption-induced changes in cyclopentane ring stereochemistry. Comparison of a wide range of thaumarchaeotal core lipid compositions revealed that the “*Ca*. Nitrosotenuis uzonensis” cultures clustered separately from other members of the NP order and the *Nitrososphaerales* (NS) order. While phylogeny generally seems to have a strong influence on GDGT distribution, our analysis of “*Ca*. Nitrosotenuis uzonensis” demonstrates that its terrestrial, higher-temperature niche has led to a lipid composition that clearly differentiates it from other NP members and that this difference is mostly driven by its high crenʹ content.

**IMPORTANCE** For *Thaumarchaeota*, the ratio of their glycerol dialkyl glycerol tetraether (GDGT) lipids depends on growth temperature, a premise that forms the basis of the widely applied TEX_86_ paleotemperature proxy. A thorough understanding of which GDGTs are produced by which *Thaumarchaeota* and what the effect of temperature is on their GDGT composition is essential for constraining the TEX_86_ proxy. “*Ca*. Nitrosotenuis uzonensis” is a moderately thermophilic thaumarchaeote enriched from a thermal spring, setting it apart in its environmental niche from the other marine mesophilic members of its order. Indeed, we found that the GDGT composition of “*Ca*. Nitrosotenuis uzonensis” cultures was distinct from those of other members of its order and was more similar to those of other thermophilic, terrestrial *Thaumarchaeota*. This suggests that while phylogeny has a strong influence on GDGT distribution, the environmental niche that a thaumarchaeote inhabits also shapes its GDGT composition.

## Abstract (precis)

For *Thaumarchaeota*, the ratio of their glycerol dialkyl glycerol tetraether (GDGT) lipids depends on growth temperature, a premise that forms the basis of the widely applied TEX_86_ paleotemperature proxy. A thorough understanding of which GDGTs are produced by which *Thaumarchaeota* and what the effect of temperature is on their GDGT composition is essential for constraining the TEX_86_ proxy. “*Ca*. Nitrosotenuis uzonensis” is a moderately thermophilic thaumarchaeote enriched from a thermal spring, setting it apart in its environmental niche from the other marine mesophilic members of its order. Indeed, we found that the GDGT composition of “*Ca*. Nitrosotenuis uzonensis” cultures was distinct from those of other members of its order and was more similar to those of other thermophilic, terrestrial *Thaumarchaeota*. This suggests that while phylogeny has a strong influence on GDGT distribution, the environmental niche that a thaumarchaeote inhabits also shapes its GDGT composition.

## INTRODUCTION

**FIG 1:**
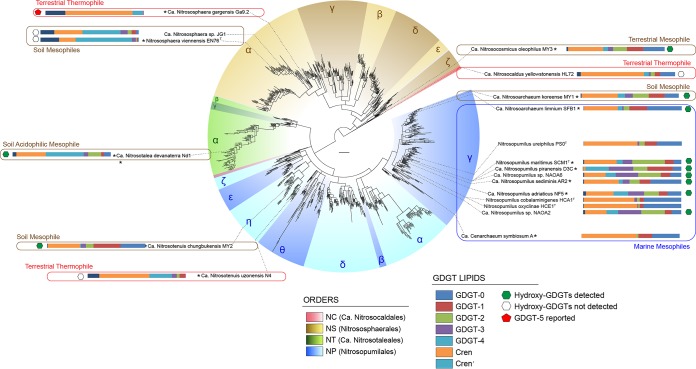
Phylogeny of select *Thaumarchaeota* that have been analyzed for core lipid composition. Phylogeny is based on *amoA* genes from cultivated and environmental AOA. Order-level lineages are indicated by four colors, major constituent subclades are indicated by Greek letters, and different shades and asterisks indicate organisms with sequenced genomes. See Fig. S1 in the supplemental material for a phylogenetic tree of all genome-sequenced *Thaumarchaeota* based on a concatenated set of universal marker genes. Horizontal bar charts represent a simplified GDGT core lipid distribution at the optimum growth temperature. Colors of bar charts are explained in the color key. Lipid data are from this study and previous studies (16, 22, 33, 34, 36–40, 79). See the text for abbreviations. Figure adapted from reference 2 with permission.

*Thaumarchaeota* are a cosmopolitan phylum of archaea. All cultured members of this phylum are ammonium oxidizers carrying out, like their bacterial counterparts, the first and rate-determining step of the nitrification process (1). Due to their high abundance and global prevalence, members of the *Thaumarchaeota* are biogeochemically important microorganisms (2–12). Based on *amoA* (2, 9), 16S rRNA (1), and concatenated gene phylogenies (13), four major orders within the phylum have been recognized (9, 13, 14): “*Candidatus* Nitrosopumilales” (NP) (also referred to as group 1.1a), “*Ca*. Nitrosotaleales” (NT) (also referred to as the SAGMCG-1 cluster or the group 1.1a-associated cluster), *Nitrososphaerales* (NS) (also referred to as group 1.1b), and “*Ca*. Nitrosocaldales” (NC) (also referred to as the ThAOA/HWCG-III cluster) (Fig. 1; see Fig. S1 in the supplemental material for a phylogenetic tree of all the genome-sequenced *Thaumarchaeota* based on concatenated universal marker genes). Many of the cultured members of the NP order are marine mesophiles, such as the *Nitrosopumilus*, *Nitrosoarchaeum*, and *Nitrosopelagicus* clades (15–19). The NT order contains three cultured members, all obligately acidophilic ammonia oxidizers (13), while the NC order contains three cultured thermophilic members of the genus “*Candidatus* Nitrosocaldus” (5, 20, 21). The NS order contains many terrestrial members, and the cultured representatives are mesophilic or moderately thermophilic and were obtained from soil, hot spring effluent, sediment, and wastewater treatment plants (22–27).

A distinct characteristic of the *Archaea* is their unique membrane lipids that set them apart from the other two domains of life. Specifically, archaeal lipids contain ether linkages between a glycerol moiety and isoprene-based alkyl chains (as opposed to the ester linkages and linear or branched alkyl chains of the *Bacteria* and *Eukarya*). Archaeal membrane lipids are mainly composed of *sn*-2,3-diphytanyl glycerol diether with two C_20_ phytanyl chains (archaeol), extended archaeol (with a C_20_ and C_25_ phytanyl chain), or *sn*-2,3-dialkyl diglycerol tetraethers with two glycerol moieties connected by two C_40_ isoprenoid chains (glycerol dialkyl glycerol tetraethers [GDGTs], which can contain 0 to 8 cyclopentane moieties [i.e., GDGT-*n*, where *n* is the number of cyclopentane moieties]) (28, 29). Thaumarchaeotal membrane core lipids identified in cultures to date (see Fig. 1 for a summary) include GDGTs ranging in the number of cyclopentane moieties from 0 to 4 (GDGT-0 to GDGT-4), hydroxy-GDGTs, crenarchaeol (cren) (containing a cyclohexane moiety) (30), an isomer of crenarchaeol (crenʹ) (31), archaeols, as well as glycerol dialkanol diethers (GDDs) and glycerol trialkyl glycerol tetraethers (GTGTs) (5, 16, 22, 32–40). To date, cren has been detected only in *Thaumarchaeota* and hence is considered to represent a specific biomarker for members of this phylum (29, 39). Indeed, the abundance of cren with intact polar headgroups has been found to covary with the *Thaumarchaeota*-specific gene abundance in the environment (41–45). Compared with the wide range of polar headgroups detected in, e.g., *Euryarchaeota* (46–50), the polar headgroups detected to date in *Thaumarchaeota* are generally based on hexose moieties (mono-, di-, and trihexose) and phosphohexose moieties (16, 32, 33, 36–38, 40).

The reason why the occurrence of cren is limited to the *Thaumarchaeota* and does not occur in other phyla of *Archaea* remains unknown. cren is characterized by the presence of an unusual cyclohexane moiety in addition to the presence of four cyclopentane moieties. Molecular modeling has revealed that this cyclohexane moiety disturbs the packing of a GDGT membrane (30), which is an important adjustment to growth temperature. Hence, the acquisition of the trait to produce a cyclohexane moiety in one of the biphytane (BP) chains of the GDGT was interpreted to represent an important step in the evolution of the *Thaumarchaeota* phylum to conquer the largest biome on Earth, the relatively cold ocean (30). Subsequently, however, cren was also identified in hot springs (51–54) and in thermophilic *Thaumarchaeota*, i.e., “*Ca*. Nitrososphaera gargensis” (NS order) (36) and *Nitrosocaldus yellowstonensis* (NC order) (33), which casts some doubt on this theory.

Some years ago, “*Ca*. Nitrosotenuis uzonensis” was cultured from a thermal spring (55). It is moderately thermophilic with an optimal growth temperature of 46°C and is the only cultured thermophile in the large, predominantly marine archaeon-dominated NP order (Fig. 1). Here we present the core and intact polar membrane lipid compositions of four replicate “*Ca*. Nitrosotenuis uzonensis” cultures grown at three different growth temperatures. We compare these with the lipid compositions of other *Thaumarchaeota* and examine further the relation between lipid composition, phylogeny, and growth temperature.

## RESULTS

### Core GDGT distribution and changes with temperature.

**FIG 2:**
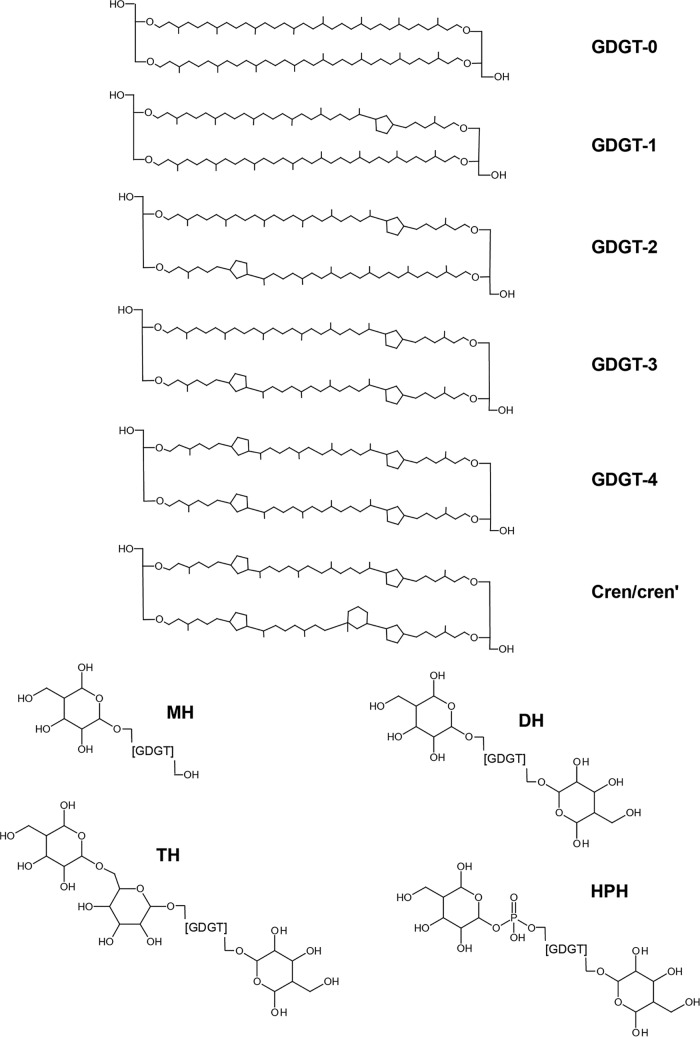
Structures of glycerol dialkyl glycerol tetraethers (GDGTs) detected in this study. Core lipids are labeled GDGT-*n*, where *n* is the number of cyclopentane moieties. cren, crenarchaeol; crenʹ, isomer of crenarchaeol. Polar headgroups are indicated (MH, monohexose; DH, dihexose; TH, trihexose; HPH, hexose/phosphohexose).

The core membrane lipids detected comprised 12 GDGTs, detected in all four replicate “*Ca*. Nitrosotenuis uzonensis” cultures at all growth temperatures. These were GDGT-0; GDGT-1 to GDGT-3, each with one isomer; GDGT-4, with two isomers; and crenarchaeol (cren) and its isomer (crenʹ, in which one cyclopentane moiety has a different stereochemistry) (31) (see Fig. 2 for structures). Previously, a Bligh-Dyer extract of “*Ca*. Nitrosotenuis uzonensis” was subjected to ether cleavage in order to examine the biphytanes (BPs) released from the GDGTs (31). The range of BPs produced were in agreement with the results of this study: in addition to a BP with no cyclopentane moieties and one with one cyclopentane moiety (data not shown), three BPs with two cyclopentane rings were produced (x, y, and I in Fig. 2 of reference 31) alongside two BPs with two cyclopentane rings and one cyclohexane ring (II and III in Fig. 2 of reference 31). The specific structural configuration of the 12 GDGTs cannot be determined from these data and would require nuclear magnetic resonance (NMR) analysis (31) or a selective *sn*2 ether cleavage protocol (56). Indeed, if both parallel and antiparallel (56) GDGT configurations are taken into consideration, the BPs detected could lead to 2 possible isomers of GDGT-0, 3 of GDGT-1, 11 of GDGT-2, 9 of GDGT-3, 15 of GDGT-4, and 18 of cren (of which cren′ would be one).

At a growth temperature of 37°C, GDGT-4 was dominant (28% ± 6% of total core GDGTs), followed by cren (25% ± 2%) and GDGT-1 (11% ± 3%) (Table 1). All other core GDGTs represented between 1 and 7% of the total. At 46°C and 50°C, cren was the dominant GDGT (51% ± 7% and 59% ± 8%, respectively), followed by GDGT-4 (16% ± 3% and 11% ± 4%, respectively) and crenʹ (12% ± 2% and 11% ± 3%, respectively), while all the remaining GDGTs accounted for between 0.4 and 6%. Eight of the core GDGTs exhibited an overall decrease in fractional abundance as the growth temperature increased (Table 1): GDGT-0 to GDGT-3, the GDGT-1 to GDGT-3 isomers, and GDGT-4. Generally, they all saw the greatest decrease in fractional abundance between temperatures of 37°C and 46°C, with only a slight or no decrease between 46°C and 50°C. The two isomers of GDGT-4 saw no overall change in fractional abundance at temperatures between 37°C and 50°C. Both cren and crenʹ increased in fractional abundance with increasing growth temperature, again with the greatest change between temperatures of 37°C and 46°C.

**TABLE 1:** Fractional abundances of core lipids from four replicate cultures of “*Ca*. Nitrosotenuis uzonensis” grown at three different temperatures^*a*^

Growth temp (°C)	Mean fractional abundance (%) ± SD											
	GDGT										cren	crenʹ
	0	1	1′	2	2′	3	3′	4	4′	4″		
37	5.3 ± 2.7	11 ± 3.3	4.8 ± 1.6	3.4 ± 0.6	2.3 ± 0.4	7.1 ± 0.4	2.9 ± 0.2	28 ± 6.3	5.3 ± 1.1	0.5 ± 0.2	25 ± 2.4	4.6 ± 1.5
46	0.4 ± 0.1	3.9 ± 0.9	1.9 ± 0.5	1.4 ± 0.2	1.5 ± 0.2	2.9 ± 0.3	2.4 ± 0.3	16 ± 2.8	6.2 ± 1.0	0.8 ± 0.2	51 ± 6.7	12 ± 2.2
50	0.5 ± 0.4	4.8 ± 1.3	2.0 ± 0.6	1.5 ± 0.2	1.4 ± 0.1	2.3 ± 0.4	1.9 ± 0.3	11 ± 3.8	4.8 ± 1.4	0.7 ± 0.1	59 ± 8.1	11 ± 3.2

a *n* = 4 for each value. Errors represent ±1 standard deviation.

### Intact polar GDGT distribution and changes with temperature.

Five different GDGT polar headgroups were detected in the “*Ca*. Nitrosotenuis uzonensis” cultures: monohexose (MH), dihexose (DH), two isomers of trihexose (TH1 and TH2), and hexose/phosphohexose (HPH) (see Fig. 2 for structures). Both of the TH isomers underwent the same fragmentation under high-performance liquid chromatography–ion trap mass spectrometry (HPLC-ITMS) (Table 2), which indicates that both sets of isomers have the same distribution of sugars around the GDGT core (as opposed to the constitutional isomers described in reference 57). Therefore, it can be assumed that they are different sugar stereoisomers. The DH-GDGT was assigned a structure with one sugar moiety on each end of the GDGT, based on comparison of the LC-ITMS^2^ fragmentation (Table 2) with those reported previously (57). The LC-ITMS^2^ fragmentation (Table 2) allowed us to further assign the two TH isomers a structure with two sugar moieties on one side and one on the other.

**TABLE 2:** MC-ITMS^2^ fragmentation of DH and TH stereoisomers with a crenarchaeol GDGT core

Polar headgroup	GDGT core	[M + NH_4_]^+^ *m/z*	MS^2^ fragment *m/z* (loss [Da])	Assignment of loss
DH1	Crenarchaeol	1,633	1,453 (−180)	Loss of sugar (C_6_H_11_O_5_) and NH_3_ from [M + NH_4_]^+^
			1,292 (−161)	Loss of C_6_H_11_O_5_ sugar from *m/z* 1,453

TH1	Crenarchaeol	1,795	1,615 (−180)	Loss of sugar (C_6_H_11_O_5_) and NH_3_ from [M + NH_4_]^+^
			1,453 (−342)	Loss of 2 sugars (C_12_H_21_O_5_) and NH_3_ from [M + NH_4_]^+^
			1,292 (−161)	Loss of C_6_H_11_O_5_ sugar from *m/z* 1,453

TH2	Crenarchaeol	1,795	1,615 (−180)	Loss of sugar (C_6_H_11_O_5_) and NH_3_ from [M + NH_4_]^+^
			1,453 (−342)	Loss of 2 sugars (C_12_H_21_O_5_) and NH_3_ from [M + NH_4_]^+^
			1,292 (−161)	Loss of C_6_H_11_O_5_ sugar from *m/z* 1,453

It should be noted that the intact polar lipids (IPLs) were examined in terms of their MS peak area response, and thus, the relative abundances reported here may not be their true relative abundances. However, this method allows for direct comparison between the “*Ca*. Nitrosotenuis uzonensis” cells cultured at different temperatures that were analyzed in this study. The headgroup distributions (Table 3) at 37°C and 46°C were quite similar, with the most dominant polar headgroup being HPH (fractional abundances of 36% ± 15% and 33% ± 3%, respectively), followed by DH (25% ± 4% and 29% ± 3%), TH1 (22% ± 3% and 23% ± 3%), and TH2 (12% ± 8% and 10% ± 5%), with the least abundant headgroup being MH (4% ± 1% and 5% ± 1%). With the increase in growth temperature from 46°C to 50°C, the headgroup distribution changed noticeably (Table 3); MH increased (from 5% ± 1% to 10% ± 5%), as did DH (from 29% ± 3% to 36% ± 8%). Indeed, DH had become the most abundant GDGT headgroup at 50°C. Considering the variability between replicate cultures, the fractional abundances of TH1 and TH2 did not exhibit an overall change with increasing temperature. The HPH GDGT headgroup decreased with the increase in growth temperature from 46°C to 50°C, from 33% ± 3% to 22% ± 9%.

**TABLE 3:** Fractional abundances of intact polar lipids from four replicate cultures of “*Ca*. Nitrosotenuis uzonensis” grown at three different temperatures^*a*^

Headgroup and core	Mean fractional abundance (%) ± SD at growth temp (°C) of:		
	37	46	50
MH			
GDGT-0	0.5 ± 0.2	0.2 ± 0.1	0.5 ± 0.2
GDGT-1	0.6 ± 0.2	0.7 ± 0.4	0.9 ± 0.4
GDGT-2	0.5 ± 0.2	0.7 ± 0.2	1.0 ± 0.5
GDGT-3	0.6 ± 0.3	0.9 ± 0.3	0.8 ± 0.4
GDGT-4	0.5 ± 0.2	0.8 ± 0.4	1.3 ± 0.9
Crenarchaeol	1.6 ± 0.5	2.1 ± 1.3	5.7 ± 2.5

Sum	4.2 ± 1.4	5.4 ± 1.3	10 ± 4.6


DH			
GDGT-0	ND	ND	ND
GDGT-1	0.6 ± 0.5	0.3 ± 0.5	0.1 ± 0.2
GDGT-2	2.8 ± 0.3	1.9 ± 0.7	2.8 ± 1.5
GDGT-3	5.0 ± 0.6	4.5 ± 0.6	5.7 ± 1.6
GDGT-4	13 ± 2.5	11 ± 1.7	13 ± 2.2
Crenarchaeol	4.0 ± 0.8	11 ± 1.1	14 ± 3.6

Sum	25 ± 3.7	29 ± 3.0	36 ± 8.6


TH1			
GDGT-0	ND	ND	ND
GDGT-1	0.8 ± 0.5	0.1 ± 0.1	ND
GDGT-2	3.4 ± 0.5	2.6 ± 0.5	1.8 ± 1.3
GDGT-3	7.1 ± 1.0	6.1 ± 1.0	5.2 ± 1.9
GDGT-4	9.9 ± 2.6	11 ± 2.1	12 ± 1.9
Crenarchaeol	1.1 ± 0.4	2.8 ± 1.4	4.9 ± 2.4

Sum	22 ± 3.2	23 ± 3.1	24 ± 5.9


TH2			
GDGT-0	ND	ND	ND
GDGT-1	ND	ND	ND
GDGT-2	ND	ND	ND
GDGT-3	2.7 ± 2.4	0.2 ± 0.4	ND
GDGT-4	8.2 ± 4.9	5.3 ± 1.9	4.3 ± 3.1
Crenarchaeol	1.3 ± 1.2	4.2 ± 3.1	3.5 ± 2.5

Sum	12 ± 8.4	9.8 ± 5.1	7.9 ± 5.5


HPH			
GDGT-0	7.0 ± 3.9	0.7 ± 0.4	0.6 ± 0.2
GDGT-1	9.2 ± 4.7	1.4 ± 0.6	1.0 ± 0.3
GDGT-2	3.8 ± 1.7	1.3 ± 0.4	0.8 ± 0.1
GDGT-3	1.8 ± 0.6	0.8 ± 0.2	0.4 ± 0.1
GDGT-4	0.2 ± 0.1	0.0 ± 0.0	0.1 ± 0.1
Crenarchaeol	14 ± 5.1	29 ± 2.5	19 ± 9.9

Sum	36 ± 15	33 ± 3.0	22 ± 9.2


[glyco]/[phosphoglyco] lipids	2.2 ± 1.6	2.0 ± 0.3	4.2 ± 2.1
wt avg no. of sugars/GDGT	2.3 ± 0.1	2.3 ± 0.1	2.2 ± 0.1

a *n* = 4 for each value. Errors represents ±1 standard deviation. ND, not detected.

**FIG 3:**
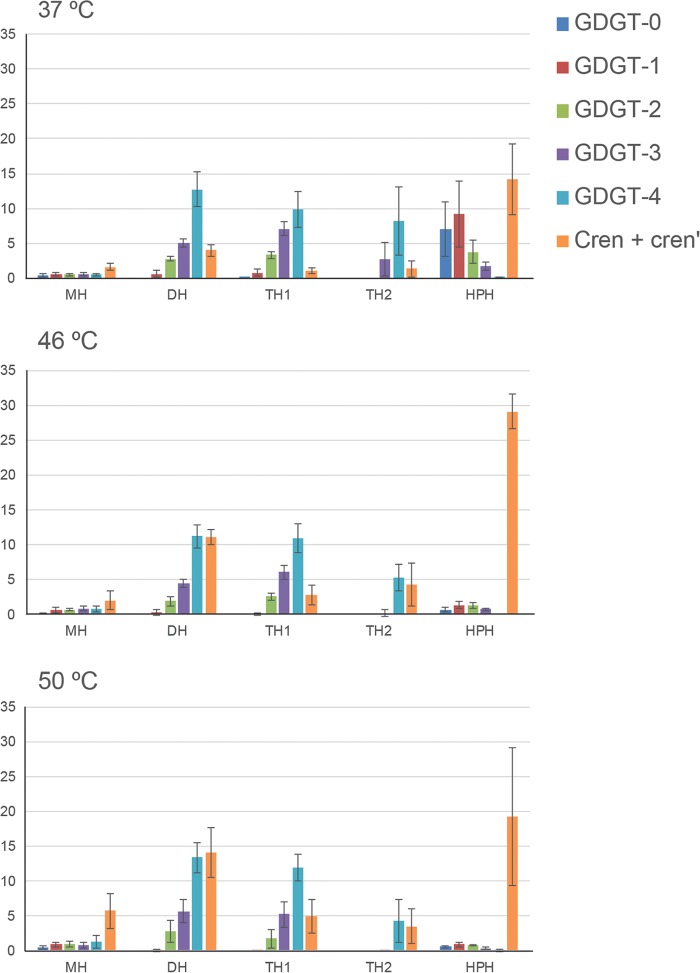
Core lipid composition per polar headgroup class for cultures of “*Ca*. Nitrosotenuis uzonensis” grown at three different temperatures. See the text for abbreviations.

A marked variability in the distribution of core lipids of each intact polar lipid was observed (Table 3 and Fig. 3). The LC-ITMS method utilized did not resolve the isomers of the GDGTs, nor did it resolve cren from crenʹ; hence, the IPL-bound GDGT cores are defined as GDGT-0 to GDGT-4 and cren. For example, cren and GDGT-0 were predominantly contained in the HPH IPL, while GDGT-4 and, to a lesser extent, GDGT-3 were predominantly found in the DH, TH1, and TH2 IPLs. Consistent with what was seen for the hydrolysis-derived core lipids, the cultures grown at 46°C and 50°C exhibited similar IPL-bound core lipid distributions, while those grown at 37°C were more distinct (Fig. 3). Overall, in the cultures grown at 46°C and 50°C, cren became more dominant for the MH and HPH IPLs and increased in DH and TH2. For the TH1 IPL, there was no change in the core lipid distribution with increasing temperature.

## DISCUSSION

“*Ca*. Nitrosotenuis uzonensis” is a moderate thermophile enriched from a thermal spring (55), which sets it apart from other cultured members of the *Nitrosopumilales* (NP) (group 1.1a), generally considered to be a predominantly marine/aquatic, mesophilic order (2). The genus *Nitrosotenuis* is the only genus within the family *Nitrosotenuaceae* within the NP, and members of this genus can be found widely distributed in soils, freshwater, hot springs, the subsurface, and activated sludge (58).

It has been reported that cren and crenʹ exist in a wide range of hot spring environments (51–54, 59–62) and in cultures of thermophilic *Thaumarchaeota*, i.e., “*Ca*. Nitrososphaera gargensis” (36) and *N. yellowstonensis* (33), contradicting the previous hypothesis that the production of cren was linked to the radiation of *Thaumarchaeota* in mesophilic environments (30). The GDGT distribution of the moderate thermophile “*Ca*. Nitrosotenuis uzonensis” further reinforces the idea that cren and crenʹ are general biomarkers for *Thaumarchaeota* rather than representing an adaptation of members of this phylum to mesophilic temperatures.

### Specific changes in “*Ca*. Nitrosotenuis uzonensis” membrane lipid composition as a response to growth temperature.

The relative abundance of the core lipids of “*Ca*. Nitrosotenuis uzonensis” varied with increasing growth temperature, with more cren and crenʹ and less GDGT-0 to GDGT-4 (including isomers). It is well established that *Thaumarchaeota* increase their cren and crenʹ proportions at higher temperatures, which explains the fundamental role of crenʹ in the sea surface temperature (SST) proxy TEX_86_ (tetraether index of tetraethers consisting of 86 carbons) (63), particularly at relatively high temperatures of >20°C. The TEX_86_ SST proxy, and its low-temperature (<15°C) and high-temperature (>15°C) versions TEX^L^_86_ and TEX^H^_86_, respectively, have been applied to temperature reconstructions in a wide range of marine and lacustrine settings (see references 29 and 64 for reviews). We calculated the TEX_86_ values for the replicate stationary “*Ca*. Nitrosotenuis uzonensis” cultures (Table 4) and applied both the core-top and mesocosm-based TEX^H^_86_ calibration described previously (65) to calculate estimated temperatures. Using the core-top TEX^H^_86_ calibration model (65), the calculated temperatures were 22°C ± 4.7°C, 32°C ± 1.7°C, and 30°C ± 2.4°C, while they were 29°C ± 3.6°C, 37°C ± 1.3°C, and 35°C ± 1.8°C, respectively, using the mesocosm TEX^H^_86_ calibration model (Table 4). The temperature calculation that gave the most similar results to the actual culture temperatures was the mesocosm TEX^H^_86_ calibration model, developed using enrichment cultures (65). However, the estimated temperatures were still on average 10°C ± 4°C lower than the actual growth temperatures, while the core-top TEX^H^_86_ calibration model gave results that were on average 16°C ± 3.4°C lower than the actual growth temperatures (Table 4). Previous studies have reported poor correlations between TEX_86_ values and temperature, and, hence, inaccurate temperature estimates, in thermophilic *Thaumarchaeota* cultures and in samples from thermal environments (33, 51, 53, 61). In this context, it is important to note that neither TEX_86_ nor TEX^H^_86_ was designed to be used in terrestrial thermal environments such as hot springs. In this context, it is also interesting to keep in mind that TEX_86_ was found to correlate with the concentration of bicarbonate, not temperature, in a range of Nevada hot springs (51). Our results now further demonstrate that TEX_86_ does not reflect well lipid membrane adaptation for thermophilic *Thaumarchaeota*. As culturing conditions other than temperature were kept constant in this study, we cannot examine the relationship that variables such as growth phase, bicarbonate concentration, ammonium oxidation rates, and pH would have on the GDGT distribution in the “*Ca*. Nitrosotenuis uzonensis” cultures.

**TABLE 4:** TEX_86_ and related values for four replicate cultures of “*Ca*. Nitrosotenuis uzonensis” grown at three different culturing temperatures^*a*^

Growth temp (°C)	Mean TEX_86_ ± SD	Mean TEX^H^_86_ temp (°C) ± SD		Avg no. of cyclopentane moieties ± SD
		Core-top calibration	Mesocosm calibration	
37	0.6 ± 0.1	22 ± 4.7	29 ± 3.6	3.1 ± 0.3
46	0.8 ± 0.05	32 ± 1.7	37 ± 1.3	3.7 ± 0.0
50	0.7 ± 0.1	30 ± 2.4	35 ± 1.8	3.7 ± 0.1

a See equations 1 to 4 and equation 6 in Materials and Methods.

It is also well established that temperature is a primary factor controlling the number of GDGT cyclopentane moieties with increasing temperature leading to an increasing number of cyclopentane moieties (63, 66–69). However, this effect was only minor for “*Ca*. Nitrosotenuis uzonensis,” as the average number of cyclopentane moieties increased from 3.1 ± 0.3 at 37°C to 3.7 ± 0.3 at 46°C and 50°C (Table 4).

As the temperature increased, specific GDGTs changed in their abundance relative to the abundance of their isomers (Table 5). With the increase in temperature from 37°C to 50°C, GDGT-2 and GDGT-2ʹ went from a distribution of 60:40 to 52:48, GDGT-3 and GDGT-3ʹ went from 71:29 to 54:46, and GDGT-4, GDGT-4ʹ, and GDGT-4″ went from 83:16:1 to 66:30:5 (Table 5). Interestingly, while the overall percentage of crenʹ increased with temperature, it remained constant relative to cren: the ratios of cren to crenʹ were 84:16 at 37°C and 85:15 at 50°C (Table 5). It has recently been revealed that crenʹ has a stereochemically different cyclopentane ring than that of cren, a difference in stereochemistry that has been postulated to have an effect on membrane fluidity, therefore playing a role in maintaining membrane homeostasis (31). In the temperature range examined for “*Ca*. Nitrosotenuis uzonensis,” crenʹ was not upregulated relative to cren as a membrane adaption to increasing temperature. However, GDGT-2ʹ was upregulated relative to GDGT-2, GDGT-3ʹ was upregulated relative to GDGT-3, and GDGT-4ʹ and GDGT-4″ were upregulated relative to GDGT-4. As explained in Results, we are not able to determine the stereochemistry of the different GDGT isomers detected in this study; however, the biphytanes (BPs) released from the GDGTs of “*Ca*. Nitrosotenuis uzonensis” grown at 46°C (31) included three different BPs with two cyclopentane rings and two BPs with two cyclopentane rings and one cyclohexane ring. Combinations of these BPs can give rise to a wide range of isomers. Here we hypothesize that the change in the composition of GDGT-2, GDGT-3, and GDGT-4 with increasing temperature represents, as per cren, changes in their cyclopentane ring stereochemistry, in order to maintain membrane homeostasis.

**TABLE 5:**
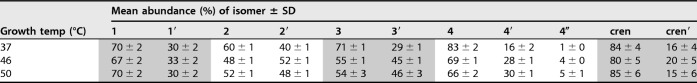
Changes in distributions of individual GDGT isomers in four replicate cultures of “*Ca*. Nitrosotenuis uzonensis” grown at three different temperatures

Whereas the core lipid compositions of the “*Ca*. Nitrosotenuis uzonensis” cultures grown at 46°C and 50°C were most similar to each other, the cultures grown at 37°C and 46°C were the most similar in terms of polar headgroup composition (Table 3 and Fig. 3). A similar observation was made previously by others (33), who noted that the core lipid and polar headgroup distributions in *Thaumarchaeota* are affected by different factors. With increasing growth temperature, the main change in the “*Ca*. Nitrosotenuis uzonensis” polar headgroup composition was that two of the smaller headgroups (MH and DH) increased in relative abundance, while one of the largest headgroups (TH2) decreased. However, when we calculated the average number of sugars per GDGT, we found no significant difference between the different growth temperatures (Table 3). To examine polar headgroup adaptions further, we calculated for each growth temperature the ratio of glycolipids to phospholipids, which was higher at 50°C (4.2 ± 2) than at 37°C and 46°C (2.2 ± 2 and 2.0 ± 0.3, respectively). Studies that describe the effect of temperature on archaeal polar headgroup composition are limited (for a review, see reference 70). The temperature-driven polar headgroup adaption reported for three strains of the *Thaumarchaeota* species Nitrosopumilus maritimus (71) (also from the NP order but a marine mesophile) was different from that seen in this study: all three *N. maritimus* strains generally decreased the relative percentage of MH lipids as the temperature increased, while the percentages of DH and HPH generally increased, and hence, in contrast to our findings, the ratio of glycolipids to phosphoglycolipids decreased as the temperature increased. However, it should be noted that the growth temperature range (18°C to 35°C) in the *N. maritimus* study was much lower. The results for “*Ca*. Nitrosotenuis uzonensis” are also different from those described previously for the *Euryarchaeota* species Thermoplasma acidophilum (thermophilic and acidophilic) (72), which was found to adapt to higher temperatures (and to lower pHs) by increasing the number of sugars in the polar headgroups. However, similar to our findings for “*Ca*. Nitrosotenuis uzonensis,” the ratio of glycolipids to phosphoglycolipids in *T. acidophilum* increased at higher temperatures. The decrease in phosphoglycolipids relative to glycolipids may relate to adaptions such as decreased proton permeability of the membrane (72–74) or could relate to stress adaption during which P-containing lipids are replaced with non-P-containing lipids in order to utilize the P for other essential cell processes. Replacement of phospholipids with nonphospholipids as a response to nutrient limitation or other stresses has been previously observed in bacteria (75), algae (76–78), and archaea (50, 74).

### What determines thaumarchaeotal lipid composition?

**FIG 4:**
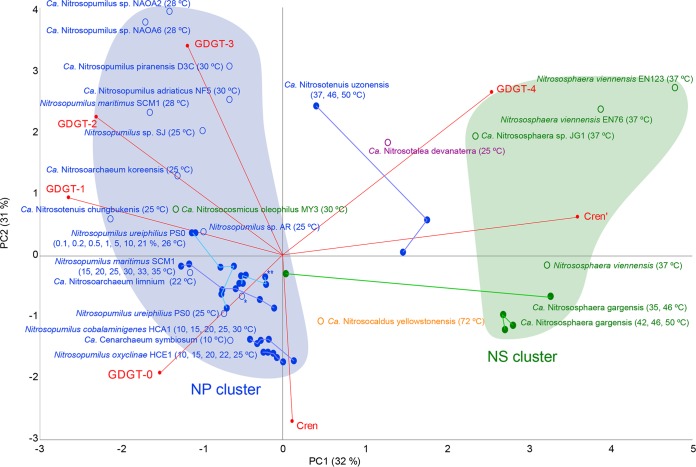
Principal-component analysis (PCA) of the simplified GDGT core lipid composition (fractional abundance) (see Table S1 in the supplemental material) for “*Ca*. Nitrosotenuis uzonensis” and other species of the *Thaumarchaeota* for which lipid composition data have been reported in the literature. For this analysis, GDGT-0 to GDGT-4 were summed with their isomers, while crenarchaeol and crenʹ were included separately, and due to their trace abundance or absence, hydroxy-GDGTs, archaeols, GDDs, and GTGTs were not included. Data are from both this study and reports in the literature (both directly reported and estimated from figures) (*n* = 56). The *Nitrosopumilales* (NP) order (group I.1a) members are in blue, *Nitrososphaerales* (NS) order (group I.1b) members are in green, the single *Nitrosotaleales* order (SAGMCG) member is in purple, and the single *Nitrosocaldales* order (HWCG) member is in orange. Experimental series are represented with filled circles and connected by a line (temperature [degrees Celsius] and/or O_2_ concentration [percent] is shown in parentheses). Filled areas represent clusters based on GDGT composition, as discussed in the text. Unfilled circle with *, Nitrosopumilus maritimus SCM1; filled circle with **, Nitrosopumilus maritimus SCM1 (0.1, 1, 5, 10, and 21%; 30°C).

We compared the core lipid composition of “*Ca*. Nitrosotenuis uzonensis” with the core lipid compositions of other thaumarchaeotal species reported in the literature (data used are listed in Table S1 in the supplemental material). To produce this simplified data set, the isomers of the GDGTs were grouped together, with the exception of cren and crenʹ, which were treated separately, while hydroxy-GDGTs were not included. First, we used principal-component analysis (PCA) (Fig. 4) to examine this data set. The first two principal components accounted for 32 and 31%, respectively, of the variability in the core lipid composition. GDGT-0 to GDGT-3 were negatively loaded on the first principal component, while GDGT-4 and crenʹ were positively loaded. cren was negatively loaded on the second principal component. The majority of the NP order members were grouped across the two negative quadrants of the first principal component, while the majority of the *Nitrososphaerales* (NS) order members were in the two positive quadrants of the first principal component, in the direction of crenʹ. The single *Nitrosotaleales* (NT) and *Nitrosocaldales* (NC) order members included in the data set were placed between the NP and NS orders (Fig. 4). There were three exceptions to this otherwise clear NP/NS separation. First, our three “*Ca*. Nitrosotenuis uzonensis” (NP order) cultures grown at different temperatures clustered between the NP and NS members. Second, a “*Ca*. Nitrososphaera gargensis” culture (moderately thermophilic; NS order), which was grown at 35°C (33), was placed within the NP order cluster. Third, “*Ca*. Nitrosocosmicus oleophilus” MY3 (NS order) (22) was placed within the NP order cluster. It should be noted that all members of the NS order that group closely together in the PCA are phylogenetically very closely related (Fig. S1). Without lipid analysis of further members of the genus *Nitrosocosmicus*, it is not possible to say whether “*Ca*. Nitrosocosmicus oleophilus” is an outlier or whether all *Nitrosocosmicus* members would group with the NP. Overall, the NP/NS cluster separation was driven by the fractional abundance of crenʹ (Fig. 4). Many of the NS members examined (e.g., “*Ca*. Nitrososphaera gargensis,” Nitrososphaera viennensis, and “*Ca*. Nitrososphaera sp.” strain JG1) contained a high percentage (14 to 29%) of crenʹ (33, 36, 40, 54, 63), while the majority of the NP members contained a lower fractional abundance (0 to 3%) of crenʹ (16, 32, 33, 37–39). This explains why the “*Ca*. Nitrosotenuis uzonensis” cultures did not cluster with the other NP order members, as all cells grown at the three different temperatures contained a relatively high fractional abundance of crenʹ (i.e., 5, 11, and 12%). It is commonly observed that the proportion of crenʹ is higher in thermophilic *Thaumarchaeota* than in mesophilic *Thaumarchaeota* (35, 36, 40), and our results suggest that this phenomenon is independent of order affiliation. The results of the PCA illustrate that while phylogeny seems to have a strong influence on GDGT distribution, environmental parameters like growth temperature can lead to inconsistencies between phylogenetic affiliation and GDGT composition, as exemplified by the moderate thermophile “*Ca*. Nitrosotenuis uzonensis” via its elevated amounts of crenʹ compared to other NP members. In this context, it should be kept in mind that factors not examined in this study have also been shown to have an effect on GDGT distribution (cf. lines linking points in Fig. 4), including O_2_ concentration, pH, and salinity (52, 71, 79).

**FIG 5:**
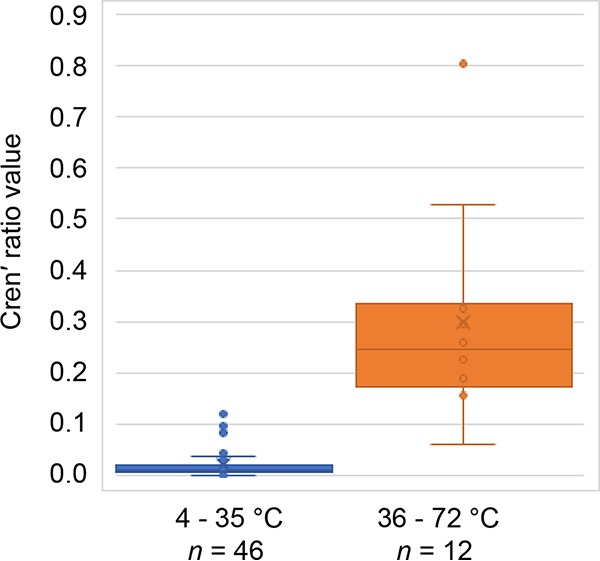
Box-and-whisker plot of the crenʹ ratios (see Table S1 in the supplemental material) in *Thaumarchaeota* grown at temperatures between 4°C and 35°C and between 36°C and 72°C. Data used to calculate the ratios were taken from this study and from the literature (Table S1).

To further examine the relationship between crenʹ and temperature across the thaumarchaeotal orders, we calculated the cren′-to-cren ratio for all known thaumarchaeotal core lipid compositions reported in the literature (Table S1) and found a significant correlation with growth temperature (Spearman *r* = 0.72; *n* = 56; *P* = <0.001). However, there appears to be a “tipping point” in the crenʹ-to-cren ratio at 35°C (Fig. 5). In the temperature range of 4°C to 35°C, the crenʹ-to-cren ratio was on average 0.02 ± 0.03, while in the range of 36°C and above, the ratio was 0.3 ± 0.2. However, it should be noted that taxon sampling is still relatively skewed in this analysis, with many members of the genus *Nitrosopumilus* being well represented and many members of the genera *Nitrosocosmicus*, *Nitrosotalea*, and *Nitrosocaldus* still awaiting lipid composition analysis. As discussed above, crenʹ has a stereochemically different cyclopentane ring than that of cren (31), which could lead to the two isomers having different effects on the fluidity of a cell membrane. It is possible that this apparent tipping point represents a *Thaumarchaeota*-wide temperature above which the different stereochemistry of the crenʹ cyclopentane ring provides a beneficial effect to the membrane. The fact that all “*Ca*. Nitrosotenuis uzonensis” cultures were grown at temperatures above this 35°C tipping point would then explain why the ratio of crenʹ to cren did not change between these growth temperatures.

A direct comparison of the intact polar lipid (IPL) composition of “*Ca*. Nitrosotenuis uzonensis” with those of other thaumarchaeotal species reported in the literature is less straightforward than for core lipids due to variability in analytical methods used between studies and the nonquantitative nature in which IPL data have often been reported. In Table 6, we summarize, in a qualitative manner, thaumarchaeotal IPL distributions reported in the literature from studies that utilized a normal-phase liquid chromatography-mass spectrometry (LCMS) method comparable to the one used in this study. The “*Ca*. Nitrosotenuis uzonensis” cultures grown at 37°C and 46°C contained HPH as the dominant polar headgroup, as has been previously reported for a range of NP species (Table 6) and also for a moderately thermophilic terrestrial NS member, “*Ca*. Nitrososphaera gargensis.” Conversely, the “*Ca*. Nitrosotenuis uzonensis” cultures grown at 50°C were dominated by DH, a characteristic IPL reported in high abundance for the NS members “*Ca*. Nitrososphaera sp.” JG1 and *N. viennensis* (40). Neither form of TH detected in “*Ca*. Nitrosotenuis uzonensis” was reported in other members of the NP (16, 37, 38), but they have been reported in the NS member *N. viennensis* (40). Previously, the lipid compositions of a range of cultured representatives of the four thaumarchaeotal orders were examined (33), and it was suggested that the core lipid composition reflects phylogenetic orders, while the polar headgroup composition reflects habitat (either terrestrial thermophiles, marine mesophiles, or soil mesophiles). Knowledge of the lipid composition of “*Ca*. Nitrosotenuis uzonensis,” which, unlike the other mesophilic members of the NP order, is moderately thermophilic (55), further confirms that environmental niche or habitat is a driver of headgroup composition. The “*Ca*. Nitrosotenuis uzonensis” cultures are more similar in headgroup composition to the three terrestrial NS members, all of which were cultured at temperatures above 35°C, than the five other NP members, all of which were cultured at temperatures below 35°C (Table 6).

**TABLE 6:** IPL compositions of *Thaumarchaeota*^*a*^

*Thaumarchaeota* member	Temp (°C)	Composition					
		MH	DH	DH-OH^*h*^	TH	PH	HPH
*Nitrosopumilales* order							
Nitrosopumilus maritimus^*b*^	28	+	+	++		Tr	+++
“*Ca*. Nitrosoarchaeum limnium” SFBI^*c*^	22		+	+		Tr	+++
Enrichment SJ^*c*^	25		+	++			+++
Enrichment AR^*c*^	25		+	+			+++
“*Ca*. Nitrosoarchaeum koreense” MY1^*d*^	25		+	++			+++
“*Ca*. Nitrosotenuis uzonensis”^*e*^	37	Tr	++		++		+++
“*Ca*. Nitrosotenuis uzonensis”^*e*^	46	Tr	++		++		+++
“*Ca*. Nitrosotenuis uzonensis”^*e*^	50	+	+++		++		++

*Nitrososphaerales* order							
“*Ca*. Nitrososphaera gargensis”^*f*^	46	Tr	+			+	+++
Nitrososphaera viennensis^*g*^	37	+	+++		++	+	+
“*Ca*. Nitrososphaera sp.” JG1^*g*^	37	Tr	+++			+	++

a Data from both this study and the literature were used. Only studies that applied a normal-phase LCMS method comparable to the one used in this study are included. Headgroups reported for only a single culture (e.g., MH+176 and DH+176) were not included. Isomers of different headgroups (e.g., TH1 and TH2) were combined. MH, monohexose; DH, dihexose; DH-OH, hydroxy dihexose; TH, trihexose; PH, phosphohexose, HPH, hexose/phosphohexose; +++, most abundant compound; ++, 50 to 100%; +, 10 to 50%; Tr, trace.

b See reference 38.

c See reference 37.

d See reference 16.

e This study.

f See reference 36.

g See reference 40.

h Hydroxy moiety on the GDGT core and not the polar headgroup (40).

The “*Ca*. Nitrosotenuis uzonensis” cultures were not found to contain IPLs with a core hydroxy-GDGT (MH-OH or DH-OH), whereas these have been detected in all other members of the NP order examined to date (16, 33, 37, 38). It should be noted that hydroxy-GDGTs were not included in the PCA (Table S1). Their absence in “*Ca*. Nitrosotenuis uzonensis” may mean that hydroxy-GDGTs are found only within specific clades of the NP order. However, hydroxy-GDGTs have also been associated with growth temperature: a decrease in temperature has been observed to lead to an increase in hydroxy-GDGTs in both thaumarchaeotal cultures (80) and environmental samples (80–83). Hence, it is possible that their absence in “*Ca*. Nitrosotenuis uzonensis” relates to the high cultivation temperatures, reflecting their thermophilic nature.

## MATERIALS AND METHODS

### Culturing.

Highly enriched “*Ca*. Nitrosotenuis uzonensis” cultures that contained no other archaea (55) were grown in medium containing (per liter) 54.4 mg KH_2_PO_4_, 74.4 mg KCl, 49.3 mg MgSO_4_·7H_2_O, 584 mg NaCl, 33.8 μg MnSO_4_, 49.4 μg H_3_BO_3_·7H_2_O, 43.1 μg ZnSO_4_·7H_2_O, 37.1 μg (NH_4_)_6_Mo_7_O_24_·4H_2_O, 97.3 mg FeSO_4_·5H_2_O, 25.0 μg CuSO_4_·2H_2_O, and 4.0 g CaCO_3_. Fresh medium batches were allowed to equilibrate for 2 weeks to ensure reaching low levels of hydrogen peroxide forming during the preparation process. Four biological replicates were grown at 37°C, 46°C, and 50°C, in 250-ml Schott flasks, in the dark. All replicates went through two 10% transfers at the respective temperatures to dilute out lipids from the inoculum culture. The cultures were fed with 1 mM NH_4_Cl (final concentration), and depletion was monitored with Nessler’s reagent. The final batches were refed multiple times, consumed 4 mM ammonium in total, and depleted ammonium before the biomass was harvested. This final biomass production step took about 8 weeks, ensuring that at the end, the vast majority of the cells produced were in stationary phase.

### Extraction.

Freeze-dried biomass lipids were extracted using a modified Bligh-Dyer procedure (84). Briefly, the biomass was treated ultrasonically three times for 10 min with a solvent mixture of methanol (MeOH), dichloromethane (DCM), and phosphate buffer (2:1:0.8, vol/vol/vol). After sonication, the combined supernatants were phase separated by adding additional DCM and buffer to a final solvent ratio of 1:1:0.9 (vol/vol/vol). The organic phase containing the intact polar lipids (IPLs) was collected, and the aqueous phase was reextracted three times with DCM. Finally, the combined extract was dried under a stream of N_2_ gas.

In order to remove the headgroups from the IPLs and to obtain the remaining core lipids, the Bligh-Dyer extract was hydrolyzed with 5% (vol/vol) HCl–MeOH by refluxing (3 h). The hydrolysate was neutralized with KOH to pH 7/8, extracted with DCM, and dried over Na_2_SO_4_.

### Core lipid analysis.

The hydrolyzed Bligh-Dyer extracts were analyzed using high-performance liquid chromatography/atmospheric-pressure chemical ionization mass spectrometry (HPLC/APCI-MS) on an Agilent 1100/Hewlett Packard 1100 MSD instrument equipped with automatic injector and HP-Chemstation software according to methods described previously (85), with the following modifications. Separation was achieved in normal phase with two Prevail Cyano columns in series (150 mm by 2.1 mm; 3 μm) with a starting eluent of hexane-propanol (99.5:0.5, vol/vol) and a flow rate of 0.2 ml min^−1^. This remained isocratic for 5 min, and thereafter, there was a linear gradient to 1.8% propanol at 45 min. The injection volume was 10 μl.

The ratios and calculations that were carried out on the core lipid data are as follows:

(1) $${\text{TEX}}_{86}=\frac{\left[\text{GDGT-2}\right]+\left[\text{GDGT-3}\right]+[\text{cren}\prime ]}{\left[\text{GDGT-}1]+[\text{GDGT-2}\right]+\left[\text{GDGT-3}\right]+[\text{cren}\prime ]}$$

(2) $${\text{TEX}}^{\text{H}}{}_{86}=log\left({\text{TEX}}_{86}\right)$$

(3) $${\text{core top TEX}}^{\text{H}}{}_{86}\text{ calibration model T}=68.4\times ({\text{TEX}}^{\text{H}}{}_{86})+38.7$$

(4) $${\text{mesocosm TEX}}^{\text{H}}{}_{86}\text{ calibration model T}=52.0\times ({\text{TEX}}^{\text{H}}{}_{86})+42.6$$

(5) cren′ratio = ([cren′])/([cren′] + [cren])

(6) $$\text{average number of cyclopentane moieties = }\frac{1\times \left(\left[\text{GDGT-}1\right]+\left[\text{GDGT-}1\prime \right]\right)+2\times \left(\left[\text{GDGT-}2\right]+\left[\text{GDGT-}2\prime \right]\right)+3\times (\left[\text{GDGT-}3\right]+\left[\text{GDGT-}3\prime \right]+4\times (\left[\text{GDGT-}4\right]+\left[\text{GDGT-}4\prime \right]+\left[\text{GDGT-}4\prime \prime \right]+\left[\text{cren}\right]+\left[\text{cren}\prime \right])}{\left[\text{GDGT-}1\right]+\left[\text{GDGT-}1\prime \right]+\left[\text{GDGT-}2\right]+\left[\text{GDGT-}2\prime \right]+\left[\text{GDGT-}3\right]+\left[\text{GDGT-}3\prime \right]+\left[\text{GDGT-}4\right]+\left[\text{GDGT-}4\prime \right]+\left[\text{GDGT-}4\prime \prime \right]+\left[\text{cren}\right]+\left[\text{cren}\prime \right]}$$

### Intact polar lipid analysis.

The Bligh-Dyer extracts were directly analyzed for IPLs. Extracts were redissolved in a mixture of hexane–2-propanol–water (72:27:1, vol/vol/vol) at a concentration of 10 mg ml^−1^. IPL extracts were analyzed by HPLC-ion trap mass spectrometry (ITMS) according to methods described previously (86), with modifications as described previously (87). The analysis was performed on an Agilent 1200 series LC instrument (Agilent, San Jose, CA), equipped with a thermostated autoinjector and column oven, coupled to an LTQ XL linear ion trap with an Ion Max source and an electrospray ionization (ESI) probe (Thermo Scientific, Waltham, MA). Separation was achieved on a LiChrospher diol column (250 by 2.1 mm, 5-μm particles; Alltech) maintained at 30°C. The following elution program was used with a flow rate of 0.2 ml min^−1^: 100% eluent A for 1 min, followed by a linear gradient to 66% eluent A–34% eluent B in 17 min, maintained for 12 min, followed by a linear gradient to 35% eluent A–65% eluent B in 15 min (where eluent A is hexane–2-propanol–formic acid–14.8 M NH_3_(aq) [79:20:0.12:0.04 {vol/vol/vol/vol}] and eluent B is 2-propanol–water–formic acid–14.8 M NH_3_(aq) [88:10:0.12:0.04 {vol/vol/vol/vol}]). The lipid extract was analyzed by an MS routine where a positive-ion scan (*m/z* 1,000 to 2,000) was followed by a data-dependent MS^2^ experiment where the base peak of the mass spectrum was fragmented (normalized collision energy [NCE] of 25, isolation width of 5.0, and activation Q of 0.175). IPLs were examined in terms of their MS peak area response. Thus, the relative abundance of the peak area does not necessarily reflect the actual relative abundance of the different IPLs; however, this method allows for comparison between the strains analyzed in this study. The peak areas were determined from extracted ion chromatograms of the [M + NH_4_]^+^ ion for each individual IPL species.

The ratios and calculations that were carried out on the intact polar lipid data are as follows:

(7) $$\text{glycolipid-to-phosphoglycolipid ratio = }\frac{\left[\text{MH-GDGTs}\right]+\left[\text{DH-GDGTs}\right]+\left[\text{TH-GDGTs}\right]}{\left[\text{HPH-GDGTs}\right]}$$

(8) $$\text{average number of sugars per GDGT}=\frac{\left[\text{MH-GDGT}\right]+2\times (\left[\text{DH-GDGT}\right]+\left[\text{HPH-GDGT}\right])+3\times \left[\text{TH-GDGT}\right]}{\left[\text{MH-GDGT}\right]+\left[\text{DH-GDGT}\right]+\left[\text{TH-GDGT}\right]+\left[\text{HPH-GDGT}\right]}$$

### Statistical analysis.

Principal-component analysis (PCA) was done using XLSTAT 2018 (Addinsoft, New York, NY). The data used for the PCA were fractional abundances (percent) that totaled 100 for each species and were not transformed further before analysis. Spearman rank correlation was done using SigmaPlot (SigmaPlot for Windows version 14; Systat Software Inc., Germany).

### Phylogenetic analyses.

An amino acid alignment of 34 universal marker genes was extracted from previously reported ammonia-oxidizing archaeon (AOA) genomes using CheckM (88). A maximum likelihood tree was constructed using IQTREE multicore version 1.6.2 (89) with 1,000 ultrafast bootstraps (90) under the best-fit model LG_F_R4, determined using ModelFinder (91) (where LG = general amino acid exchange matrix [92], F = empirical amino acid frequencies from the data, and R4 = rate heterogeneity calculated under the FreeRate model [93, 94] with four categories).

## Supplementary Material

Supplemental file 1
