# Supplementary material for: Membrane Lipid Composition of the Moderately Thermophilic Ammonia-Oxidizing Archaeon “Candidatus Nitrosotenuis uzonensis” at Different Growth Temperatures
Source: Appl Environ Microbiol. 2019 Oct 1;85(20):e01332-19. doi: 10.1128/AEM.01332-19 (PMC6805073; doi:10.1128/AEM.01332-19)
Supplement: Supplemental file 1 [file AEM.01332-19-s0001.pdf]

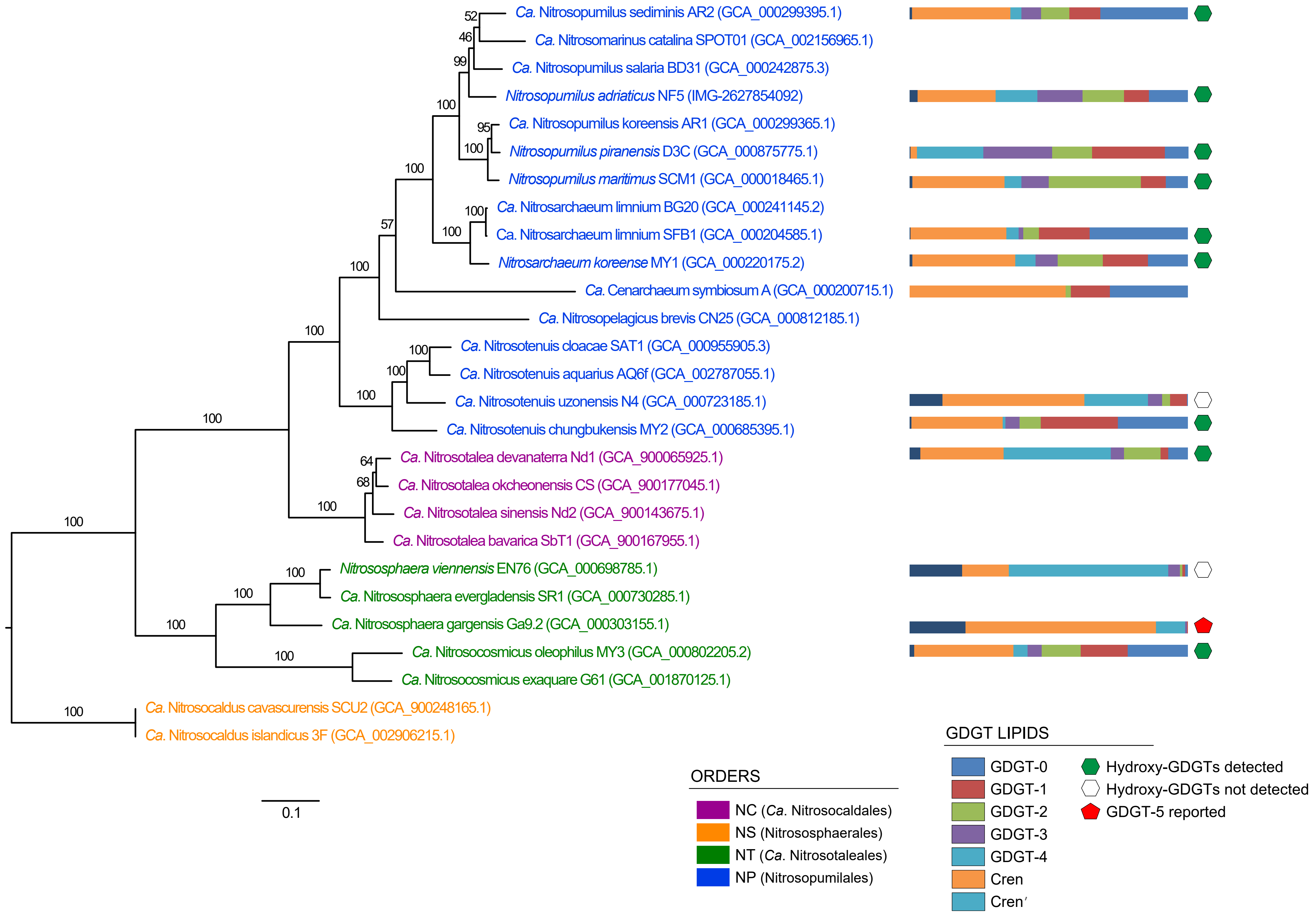

Ca. Nitrosopumilus sediminis AR2 (GCA_000299395.1)
Ca. Nitrosomarinus catalina SPOT01 (GCA_002156965.1)
Ca. Nitrosopumilus salaria BD31 (GCA_000242875.3)
Nitrosopumilus adriaticus NF5 (IMG-2627854092)
Ca. Nitrosopumilus koreensis AR1 (GCA_000299365.1)
Nitrosopumilus piranensis D3C (GCA_000875775.1)
Nitrosopumilus maritimus SCM1 (GCA_000018465.1)
Ca. Nitrosarchaeum limnium BG20 (GCA_000241145.2)
Ca. Nitrosarchaeum limnium SFB1 (GCA_000204585.1)
Nitrosarchaeum koreense MY1 (GCA_000220175.2)
Ca. Cenarchaeum symbiosum A (GCA_000200715.1)
Ca. Nitrosopelagicus brevis CN25 (GCA_000812185.1)
Ca. Nitrosotenuis cloacae SAT1 (GCA_000955905.3)
Ca. Nitrosotenuis aquarius AQ6f (GCA_002787055.1)
Ca. Nitrosotenuis uzonensis N4 (GCA_000723185.1)
Ca. Nitrosotenuis chungbukensis MY2 (GCA_000685395.1)
Ca. Nitrosotalea devanaterra Nd1 (GCA_900065925.1)
Ca. Nitrosotalea okcheonensis CS (GCA_900177045.1)
Ca. Nitrosotalea sinensis Nd2 (GCA_900143675.1)
Ca. Nitrosotalea bavarica SbT1 (GCA_900167955.1)
Nitrososphaera viennensis EN76 (GCA_000698785.1)
Ca. Nitrososphaera evergladensis SR1 (GCA_000730285.1)
Ca. Nitrososphaera gargensis Ga9.2 (GCA_000303155.1)
Ca. Nitrosocosmicus oleophilus MY3 (GCA_000802205.2)
Ca. Nitrosocosmicus exaquare G61 (GCA_001870125.1)
Ca. Nitrosocaldus cavascurensis SCU2 (GCA_900248165.1)
Ca. Nitrosocaldus islandicus 3F (GCA_002906215.1)
0.1
ORDERS
NC (Ca. Nitrosocaldales)
NS (Nitrososphaerales)
NT (Ca. Nitrosotaleales)
NP (Nitrosopumilales)
GDGT LIPIDS
GDGT-0
GDGT-1
GDGT-2
GDGT-3
GDGT-4
Cren
Cren′
Hydroxy-GDGTs detected
Hydroxy-GDGTs not detected
GDGT-5 reported

Figure S1. Maximum likelihood phylogenetic tree of all genome-sequenced Thaumarchaeota based on a concatenated alignment of 34 universal marker genes. The tree was calculated with IQTREE. Ultrafast bootstrap values (UFBoot; n= 1000 replicates) are indicated on the branches. Bar indicates number of amino acid changes per site. Horizontal bar charts represent simplified GDGT core lipid distribution at optimum growth temperature. Colors of bar charts are explained in the color legend. A green hexagon indicates detection of hydroxy-GDGTs, while a white hexagon represents report that hydroxy-GDGTs were not detected. Red pentagon represents detection of GDGT-5. Lipid data from this study and references 1–10. See text for abbreviations.

Supplement Table 1. Fractional abundance of core lipids composition (relative percent) for species of Thaumarchaeota reported in the literature (both directly reported and estimated from published figures). For Cren' ratio See formula 5 in Material and Methods.

	Temp	GDGT-0	GDGT-1	GDGT-2	GDGT-3	GDGT-4	Cren	Cren′	Cren' ratio
**Nitrosopumilales order**									
Nitrosopumilus maritimus SCM1[a]	**28**	13	8	10	4	0	65	0	0.00
Nitrosopumilus maritimus SCM1[b]	**28**	8	9	33	10	6	33	1	0.03
Nitrosopumilus maritimus SCM1[c]	**15**	28	15	9	4	0	44	0.2	0.00
Nitrosopumilus *maritimus* SCM1[c]	**20**	23	14	11	4	0	48	0.2	0.00
Nitrosopumilus maritimus SCM1[c]	**25**	21	12	7	5	0	54	0.4	0.01
Nitrosopumilus maritimus SCM1[c]	**30**	13	10	9	4	0	63	0.6	0.01
Nitrosopumilus maritimus SCM1[c]	**33**	6	6	12	4	0	72	0.6	0.01
Nitrosopumilus maritimus SCM1[c]	**35**	6	5	11	3	0	74	0.8	0.01
Nitrosopumilus ureiphilius PS0[c]	**25**	25	13	6	2	0	54	0.3	0.01
Nitrosopumilus cobalaminigenes HCA1[c]	**10**	59	4	2	2	0	33	0.3	0.01
Nitrosopumilus cobalaminigenes HCA1[c]	**15**	58	3	2	2	0	35	0.3	0.01
Nitrosopumilus cobalaminigenes HCA1[c]	**20**	50	4	2	2	0	41	0.3	0.01
Nitrosopumilus cobalaminigenes HCA1[c]	**25**	38	5	3	3	0	52	0.4	0.01
Nitrosopumilus cobalaminigenes HCA1[c]	**30**	27	4	2	1	0	65	0.5	0.01
Nitrosopumilus oxyclinae HCE1[c]	**10**	57	3	1	1	0	37	0.1	0.00
Nitrosopumilus oxyclinae HCE1[c]	**15**	54	3	1	2	0	40	0.2	0.00
Nitrosopumilus oxyclinae HCE1[c]	**20**	48	3	2	2	0	45	0.2	0.01
Nitrosopumilus oxyclinae HCE1[c]	**22**	44	3	1	1	0	50	0.2	0.00
Nitrosopumilus oxyclinae HCE1[c]	**25**	39	4	1	1	0	55	0.3	0.01
Nitrosopumilus maritimus SCM1 0.1 % O_2[c]	**30**	4	3	17	5	0	70	1	0.02
Nitrosopumilus maritimus SCM1 1 % O_2[c]	**30**	4	3	16	4	0	71	1	0.02
Nitrosopumilus maritimus SCM1 5 % O_2[c]	**30**	8	7	15	4	0	65	1	0.01
Nitrosopumilus maritimus SCM1 10 % O_2[c]	**30**	9	7	13	4	0	66	1	0.01
Nitrosopumilus maritimus SCM1 21 % O_2[c]	**30**	10	8	12	4	0	64	1	0.01
Nitrosopumilus ureiphilius PS0 0.1 % O_2[c]	**26**	10	6	27	3	0	54	0.4	0.01
Nitrosopumilus ureiphilius PS0 0.2 % O_2[c]	**26**	9	7	26	3	0	54	0.4	0.01
Nitrosopumilus ureiphilius PS0 0.5 % O_2[c]	**26**	10	8	18	3	0	60	1	0.01
Nitrosopumilus ureiphilius PS0 1 % O_2[c]	**26**	2	9	16	3	0	60	1	0.01
Nitrosopumilus ureiphilius PS0 5 % O_2[c]	**26**	15	10	13	2	0	59	0.4	0.01
Nitrosopumilus ureiphilius PS0 10 % O_2[c]	**26**	16	11	11	2	0	59	0.4	0.01
Nitrosopumilus ureiphilius PS0 21 % O_2[c]	**26**	20	12	8	2	0	59	0.3	0.00

Ca. Nitrosopumilus sp. NAOA2[b]	**28**	10	7	24	24	12	22	2	0.08
Ca. Nitrosopumilus maritimus NAOA6[b]	**28**	11	10	23	23	10	22	1	0.04
Ca. Nitrosopumilus piranensis D3C[b]	**30**	9	8	24	13	23	22	2	0.08
Ca. Nitrosopumilus adriaticus NF5[b]	**30**	14	9	15	16	15	28	3	0.10
Ca. Cenarchaeum symbiosum[d]	**10**	28	14	2.0	0	0	56	0	0.00
Ca. Nitrosoarchaeum limnium SFBI[e]	**22**	35	18	5.5	1.6	4.5	34	0.4	0.01
Nitrosopumilus *sp.* SJ[e]	**25**	26	9.4	14	14	12	24	1	0.04
Nitrosopumilus *sp.* AR[e]	**25**	31	11	10	6.8	4.0	35	0.6	0.02
Ca. Nitrosoarchaeum koreense MY1[f]	**25**	14	16	16	7.6	7.3	36	1.1	0.03
Ca. Nitrosotenuis uzonensis N4[g]	**37**	5	16	5	10	34	25	5	0.17
Ca. Nitrosotenuis uzonensis N4[g]	**46**	0.4	6	3	5	23	51	12	0.19
Ca. Nitrosotenuis uzonensis N4[g]	**50**	1	7	3	4	17	59	11	0.16
Ca. Nitrosotenuis chungbukensis MY2[h]	**25**	25	28	7.5	5.1	0.8	33	0.6	0.02
**Nitrososphaerales order**									
Ca. Nitrososphaera gargensis[i]	**42**	0.3	0.4	0.5	1.0	3.2	69	24	0.26
Ca. Nitrososphaera gargensis[i]	**46**	0.1	0.1	0.2	0.4	10	65	19	0.23
Ca. Nitrososphaera gargensis[i]	**50**	0.1	0.1	0.2	0.4	5.6	70	21	0.23
Ca. Nitrososphaera gargensis[b]	**35**	15	7	8	4	8	56	2	0.03
Ca. Nitrososphaera gargensis[b]	**46**	1	1	1	1	6	59	30	0.34
Nitrososphaera viennensis[j]	**37**	0.3	0.3	0.5	1.5	20	55	23	0.29
Nitrososphaera viennensis EN123[b]	**37**	0.5*	0.5*	1	3	60	7	29	0.81
Nitrososphaera viennensis EN76[b]	**37**	1	1	1	4	58	17	19	0.53
Ca. Nitrososphaera sp. JG1[j]	**37**	2.9	4.3	4.0	7.2	39	29	14	0.33
Ca. Nitrosocosmicus oleophilus MY3[k]	**30**	22	17	14	5.3	5.3	36	1.6	0.04
**Nitrosocaldales order**									
Ca. Nitrosocaldus yellowstonensis[b]	**72**	15	6	4	2	5	65	4	0.06
**Nitrosotaleales order**									
Ca. Nitrosotalea devanaterra[b]	**25**	7	3	13	5	39	30	4	0.12

*Estimated values (reported as <1). a = (1); b = (2); c = (3) ; d = (4); e = (5); f = (6); g = this study; h = (7); i = (8); j = (9); k = (10).

1. Schouten S, Hopmans EC, Baas M, Boumann H, Standfest S, Koenneke M, Stahl DA, Sinninghe Damsté JS. 2008. Intact membrane lipids of “*Candidatus* Nitrosopumilus maritimus,” a cultivated representative of the cosmopolitan mesophilic group I crenarchaeota. Appl Environ Microbiol 74:2433–2440.

2. Elling FJ, Könneke M, Nicol GW, Stieglmeier M, Bayer B, Spieck E, de la Torre JR, Becker KW, Thomm M, Prosser JI, Herndl GJ, Schleper C, Hinrichs K-U. 2017. Chemotaxonomic characterisation of the thaumarchaeal lipidome. Environ Microbiol 19:2681–2700.

3. Qin W, Carlson LT, Armbrust EV, Devol AH, Moffett JW, Stahl DA, Ingalls AE. 2015. Confounding effects of oxygen and temperature on the TEX86 signature of marine Thaumarchaeota. PNAS 112:10979–10984.

4. Sinninghe Damsté JS, Rijpstra WIC, Hopmans EC, Prahl FG, Wakeham SG, Schouten S. 2002. Distribution of Membrane Lipids of Planktonic Crenarchaeota in the Arabian Sea. Appl Environ Microbiol 68:2997–3002.

5. Pitcher A, Hopmans EC, Mosier AC, Park S-J, Rhee S-K, Francis CA, Schouten S, Sinninghe Damsté JS. 2011. Core and intact polar glycerol dibiphytanyl glycerol tetraether lipids of ammonia-oxidizing archaea enriched from marine and estuarine sediments. Appl Environ Microbiol 77:3468–3477.

6. Jung M-Y, Park S-J, Min D, Kim J-S, Rijpstra WIC, Damsté JSS, Kim G-J, Madsen EL, Rhee S-K. 2011. Enrichment and Characterization of an Autotrophic Ammonia-Oxidizing Archaeon of Mesophilic Crenarchaeal Group I.1a from an Agricultural Soil. Appl Environ Microbiol 77:8635–8647.

7. Jung M-Y, Park S-J, Kim S-J, Kim J-G, Sinninghe Damsté JS, Jeon CO, Rhee S-K. 2014. A mesophilic, autotrophic, ammonia-oxidizing archaeon of thaumarchaeal group I.1a cultivated from a deep oligotrophic soil horizon. Appl Environ Microbiol 80:3645–3655.

8. Pitcher A, Rychlik N, Hopmans EC, Spieck E, Rijpstra WIC, Ossebaar J, Schouten S, Wagner M, Damsté JSS. 2010. Crenarchaeol dominates the membrane lipids of Candidatus Nitrososphaera gargensis, a thermophilic group I.1b Archaeon. ISME J 4:542–552.

9. Sinninghe Damsté JS, Rijpstra WIC, Hopmans EC, Jung M-Y, Kim J-G, Rhee S-K, Stieglmeier M, Schleper C. 2012. Intact Polar and Core Glycerol Dibiphytanyl Glycerol Tetraether Lipids of Group I.1a and I.1b Thaumarchaeota in Soil. Appl Environ Microbiol 78:6866–6874.

10. Jung M-Y, Kim J-G, Sinninghe Damsté JS, Rijpstra WIC, Madsen EL, Kim S-J, Hong H, Si O-J, Kerou M, Schleper C, Rhee S-K. 2016. A hydrophobic ammonia-oxidizing archaeon of the Nitrosocosmicus clade isolated from coal tar-contaminated sediment. Environ Microbiol Rep 8:983–992.
